# Histopathological Assessment of IFI6- and RSAD2-DNA Aptamers in Oral Squamous Cell Carcinoma: A Preliminary Study Based on *In Silico* Analysis

**DOI:** 10.3390/biomedicines14081684

**Published:** 2026-07-27

**Authors:** Danial Qasim Butt, Maaz Anwer Memon, Masitah Hayati Harun, Shazana Hilda Shamsuddin, Nur Asyilla Binti Che Jalil, Saidi Jaafar, Teffanie Arputheraj, Basaruddin Ahmad

**Affiliations:** 1 Oral Medicine and Oral Pathology Unit, School of Dental Sciences, Universiti Sains Malaysia, Kubang Kerian 16150, Kelantan, Malaysia; danial_qasim.scd@stmu.edu.pk (D.Q.B.); masitahh@usm.my (M.H.H.); 2 Oral Pathology Department, Shifa College of Dentistry, Shifa Tameer-E-Millat University, Islamabad 44000, Pakistan; 3 Department of Pathology, School of Medical Sciences, Universiti Sains Malaysia, Health Campus, Kubang Kerian 16150, Kelantan, Malaysia; asyilla@usm.my; 4 Basic Sciences Unit, School of Dental Sciences, Universiti Sains Malaysia, Kubang Kerian 16150, Kelantan, Malaysia; saidijaafar@usm.my; 5 Biogenes Technologies Sdn Bhd, Serdang 43400, Selangor, Malaysia; teffanie@biogenestech.com; 6 Biostatistics Unit, School of Dental Sciences, Universiti Sains Malaysia, Kubang Kerian 16150, Kelantan, Malaysia

**Keywords:** *in silico* DNA aptamer, IFI6, RSAD2, OSCC, molecular docking, molecular dynamics simulation

## Abstract

**Introduction:** DNA aptamers are single-stranded nucleic acids capable of selectively binding target proteins and have emerged as potential alternatives to antibodies in molecular diagnostics. This study aimed to develop and characterize *in silico* designed DNA aptamers targeting Interferon alpha-inducible protein 6 (IFI6) and Radical S-adenosyl-L-methionine domain-containing protein 2 (RSAD2) for oral squamous cell carcinoma (OSCC). **Methods:** Genomic transfer RNA sequences from *Homo sapiens*, *Mus musculus*, and *Escherichia coli* were computationally truncated and optimized to generate DNA aptamer candidates ranging from 35–50 mers. Secondary and tertiary structures were generated using Mfold and RNAComposer, followed by molecular docking with AutoDock Vina and molecular dynamics simulations in GROMACS to evaluate docking interactions and structural behavior comparatively. Selected DNA aptamer candidates were synthesized and evaluated by aptahistochemistry (AHC) to qualitatively explore preliminary tissue reactivity in formalin-fixed paraffin-embedded OSCC tissues under optimized conditions. **Results:** Molecular docking demonstrated favorable comparative docking scores ranging from −15.6 to −18.7 kcal/mol. RMSD analysis demonstrated that selected IFI6 and RSAD2 DNA aptamer–protein complexes reached a plateau with relatively small fluctuations following maximum RMSD values. In contrast, RMSF analysis identified increased flexibility predominantly within loop regions. Cross-reactivity analysis explored preliminary target selectivity, with no interactions observed within the selected target-binding regions. During AHC optimization, the 50-IFI6 and 45-RSAD2 DNA aptamer candidates exhibited cytoplasmic brown granular staining in >50% of OSCC tumor cells under the optimized experimental conditions. **Conclusions:** The findings support the applicability of an integrated *in silico* workflow for generating structurally optimized DNA aptamer candidates and provide preliminary proof-of-concept for their exploratory histopathological application in OSCC.

## 1. Introduction

Oral squamous cell carcinoma (OSCC), the predominant subtype of head and neck squamous cell carcinoma (HNSCC), remains an aggressive and immune-influenced malignancy with persistently poor outcomes [1]. According to the latest GLOBOCAN 2022 estimates, head and neck cancer accounts for approximately 946,000 new cases and 478,000 deaths worldwide annually, highlighting its substantial global health burden [2].

DNA aptamers are single-stranded oligonucleotides that fold into specific three-dimensional structures, enabling selective binding to target molecules, comparable to antibody–antigen interactions [3]. The aptamers have shown significant promise in targeting cancer-associated antigens and identifying biomarkers involved in tumorigenesis [4,5,6,7,8].

Traditionally, DNA aptamers are generated through the Systematic Evolution of Ligands by Exponential Enrichment (SELEX) technique, which involves iterative selection and amplification of high-affinity aptamers from a vast oligonucleotide library [9]. However, SELEX is a labor-intensive and time-consuming technique [10]. Recent advancements *in silico* biocomputational methods offer a more efficient and cost-effective alternative, leveraging genomic data to design aptamers with high selectivity for cancer antigens [11].

Interferon alpha-inducible protein 6 (IFI6) and Radical S-adenosyl-L-methionine domain-containing protein 2 (RSAD2) are reported as potential biomarkers found in cancers like oral squamous cell carcinoma (OSCC), breast, lung, gastric, colorectal, and esophageal squamous cell carcinoma (ESCC) identified via immunohistochemistry (IHC) [12,13,14,15,16,17]. Both proteins have been implicated in tumor progression, evasion of apoptosis, immune modulation, cellular proliferation, and metastatic behavior within the tumor microenvironment, suggesting important biological roles in cancer pathogenesis. Furthermore, the predominantly cytoplasmic expression patterns of IFI6 and RSAD2, together with their reported overexpression in malignant tissues and limited expression in healthy tissues, make them attractive molecular targets for the development of tissue-based detection probes [15,16]. However, traditional polyclonal antibodies used in IHC may exhibit cross-reactivity and batch-to-batch variability, potentially resulting in inconsistent staining patterns and off-target detection [18]. In contrast, DNA aptamers are non-immunogenic, highly specific, and thus have been used in aptamer-based assays like aptahistochemistry (AHC) applications in tissues [19]. To the best of our knowledge, no previous studies have reported the development and tissue-based detection of OSCC-associated protein biomarkers using DNA aptamers through AHC.

Therefore, the present study was designed as a proof-of-concept investigation to evaluate the feasibility of generating DNA aptamer candidates against IFI6 and RSAD2 proteins through an integrated computational workflow and to perform preliminary histopathological assessment using AHC optimization. The specific objectives were to design and characterize *in silico* DNA aptamer candidates targeting IFI6 and RSAD2 proteins and to assess their tissue reactivity in OSCC through AHC optimization.

## 2. Materials and Methods

The study protocol was approved by the Human Research Ethics Committee, Universiti Sains Malaysia (HREC) (USM/JEPeM/KK/23030256). The study was divided into two stages: (1) Designing and Characterization of DNA aptamers, and (2) AHC optimization of DNA aptamers. This study used archival-diagnosed Formalin-Fixed Paraffin-Embedded (FFPE) OSCC tissue blocks from the Oral Pathology Laboratory, School of Dental Sciences, and the Pathology Laboratory, School of Medical Sciences, Hospital Universiti Sains Malaysia (HUSM). All *in silico* analyses were performed on a Windows 10 (64-bit) workstation equipped with an Intel^®^ Core™ i9-9900KF CPU (3.60 GHz) and 16.0 GB RAM at the School of Dental Sciences, Universiti Sains Malaysia (USM). The workflow incorporated multiple specialized software and databases [20]. Transfer RNA (tRNA) sequences were obtained from the Genomic tRNA Database (GtRNAdb). Secondary structures of aptamers were predicted using Mfold version 3.6, while three-dimensional models were generated in RNAComposer. RNA-to-DNA conversion and structural modifications were performed in PyMOL (v3.0.3) and AutoDock Tools (v1.5.6). Molecular docking was conducted with AutoDock Vina (qvina2). Molecular dynamics (MD) simulations were performed using GROMACS (v2020 rcl).

### 2.1. Designing of In Silico DNA Aptamer

Three genomic primary tRNA sequences of different lengths (mers) were obtained from the Genomic tRNA Database (GtRNAdb): *Homo sapiens* (HS, 72-mers), *Mus musculus* (MM, 72-mers), and *Escherichia coli* (EC, 76-mers) [21]. Each was truncated into five candidates (35–50 mers): 35HS, 45MM, 50HS, 35MM, and 50EC. The selection of genomic tRNA sequences was based on their highly conserved, naturally evolved secondary-structure organization rather than on sequence homology to the target proteins. Transfer RNA molecules possess stable stem–loop and hairpin conformations that serve as templates for generating nucleic acid ligands. In the present study, genomic tRNA sequences with high tRNA prediction scores (>80) were selected as structural templates to preserve folding fidelity and thermodynamic stability during truncation. Truncation was guided by maintaining a balanced GC/AT composition, consistent with the general compositional principles described by Chargaff’s parity rule, while preserving stem–loop regions to support secondary-structure integrity [22]. The truncation principles included maintaining GC and AT composition within ±5%, preserving the loop region containing the GTAC sequence in the genomic tRNA template, removing redundant nucleotides to improve structural stability, and symmetrically deleting paired bases to preserve stem orientation. As genomic sequences obtained from GtRNAdb were used in the present study, the GTAC sequence within the loop region represents the genomic template sequence rather than the canonical TΨC loop of mature post-transcriptionally modified tRNA. The genomic sequence was appropriate for this study because it served as an unmodified structural template for synthetic DNA aptamer design, using only the standard DNA nucleotides A, T, G, and C rather than the modified nucleotides present in mature tRNA. Secondary (2D) structures were generated using the Mfold version 3.6 under thermodynamic conditions of 37 °C, 1.0 M Na^+^, and 0 M Mg^2+^ [23]. Tertiary (3D) structures were modeled via RNAComposer and used as templates for DNA conversion [24]. The ssRNA was imported into PyMOL 3.0 for conversion into ssDNA aptamers [25]. Uracil residues were replaced with thymine, and the 2′-ribose hydroxyl groups were removed using PyMOL command prompts to generate DNA aptamer structural models. These structures served as the initial templates for molecular docking. Subsequent energy minimization and molecular dynamics simulations of the docked complexes were performed as described in the molecular dynamics simulation section. Non-polar hydrogens were deleted to retain chemically relevant interactions. The resulting ssDNA structures were refined in AutoDock Tools 4 (v1.5.6) by restricting selected C–O and C–N rotatable bonds to generate semi-flexible conformations. This approach was utilized to reduce excessive conformational freedom associated with the relatively large size and structural flexibility of the DNA aptamer candidates, thereby facilitating comparative molecular docking analyses under standardized computational conditions [26]. Aptamer candidates 35HS and 50HS, designed against IFI6, were designated as 35-IFI6 and 50-IFI6. Candidates 35MM and 50EC, generated for RSAD2, were designated as 35-RSAD2 and 50-RSAD2. However, the 45MM candidate was applied to both IFI6 and RSAD2 for comparative observation and analysis, and was therefore designated as 45-IFI6 when used against IFI6 and 45-RSAD2 when used against RSAD2.

### 2.2. Characterization of Molecular Binding Interactions

#### 2.2.1. Molecular Docking and Molecular Dynamics Simulation

Molecular docking simulations were performed using AutoDock Vina (QVina2), targeting selected docking or target-binding regions of IFI6 (N-terminal; residues 1–50) and RSAD2 (C-terminal; residues 170–361). The AlphaFold Protein Structure Database (AlphaFold v2) models corresponding to the canonical human protein sequences of IFI6 (UniProt accession: P09912) and RSAD2 (UniProt accession: Q8WXG1) were used for molecular docking. The selected docking or target-binding regions were chosen based on the antigenic sequence and antibody-associated sequence annotations available in the UniProt database, together with the immunogen regions of the commercially available primary antibodies (IFI6: PA5-99824; RSAD2: PA5-106337; Invitrogen, Waltham, MA, USA). This strategy was adopted to maintain consistency between the computational docking analyses and the subsequent tissue-based experiments and was not intended to define experimentally resolved antibody epitopes. The mean per-residue confidence (pLDDT) scores of the selected target-binding regions were 52.90 (range: 26.22–69.00) for IFI6 residues 1–50 and 95.31 (range: 68.00–98.81) for RSAD2 residues 170–361. Structural confidence was considered during the interpretation of the molecular docking results but was not used as the primary criterion for selecting the target-binding regions. The protein models were prepared in AutoDock Tools 1.5.6 by the addition of polar hydrogens and partial charges before molecular docking analysis. Grid boxes with a spacing of 0.375 Å were centered on the selected target-binding regions of each protein. Consequently, molecular docking was performed using a targeted docking strategy rather than blind docking. Molecular docking analyses were performed to evaluate the relative interaction profiles of the DNA aptamer candidates with their respective target proteins. The resulting docking scores and interaction analyses were subsequently used as an initial comparative screening step for DNA aptamer candidates before molecular dynamics simulations and subsequent assessment of tissue reactivity by aptahistochemistry. Because the comparative docking scores among the evaluated DNA aptamer candidates showed only a relatively narrow range, molecular docking alone provided limited discrimination for candidate selection. Therefore, AutoDock Vina was employed as one component of an integrated computational workflow, and the docking scores were interpreted solely as relative indicators of candidate binding poses and interaction characteristics rather than as quantitative estimates of absolute thermodynamic binding free energies. Final candidate prioritization was based on the integrated interpretation of molecular docking, molecular dynamics simulations, cross-reactivity analysis, and exploratory tissue reactivity assessment rather than on docking scores alone. Binding poses and interaction analyses were visualized and recorded using PyMOL.

Molecular dynamics (MD) simulations were performed in GROMACS (2020 rc1) using the AMBER94 force field [27]. Topology files for the aptamer–protein complexes were generated using the pdb2gmx module in GROMACS with the AMBER94 force field, which assigns force-field parameters for bonded and non-bonded interactions, including atom types, partial charges, bond lengths, bond angles, dihedral angles, and van der Waals and electrostatic interaction terms. The AMBER94 force field was selected because it contains established parameters for both proteins and nucleic acids and has been extensively applied in molecular dynamics simulations of biomolecular complexes, including DNA–protein interactions. Its compatibility with nucleic acid systems and implementation within GROMACS make it suitable for evaluating the structural stability and flexibility of aptamer–protein complexes [20]. The generated topology files (.top and .itp) were subsequently used for solvation, ion addition, energy minimization, equilibration, and production MD simulations. The complexes were placed in cubic simulation boxes with a minimum distance of 3.0 nm between the solute and the box edge and solvated using the SPC216 water model. Na^+^ and Cl^−^ ions were added to neutralize the overall charge of each aptamer–protein complex. Each aptamer–protein complex was placed in a neutralized cubic box and subjected to energy minimization using the steepest descent algorithm with a maximum force threshold of 1000.0 kJ/mol/nm for up to 50,000 steps to remove steric clashes and optimize system geometry. Subsequently, the systems were equilibrated under NVT (constant number of particles, volume, and temperature) conditions for 100 ps, followed by NPT (constant number of particles, pressure, and temperature) equilibration for an additional 100 ps with position restraints applied to the solute to stabilize temperature and pressure. Production molecular dynamics simulations were then performed without restraints for 5000 ps (5 ns; 2,500,000 steps) using the gmx mdrun module. The root mean square deviation (RMSD) analysis was performed using the initial docked aptamer–protein complex as the reference structure after least-squares fitting to the protein backbone to monitor overall structural deviations throughout the molecular dynamics trajectory. Root mean square fluctuation (RMSF) was subsequently performed to evaluate local flexibility within the DNA aptamer candidates, where higher RMSF values indicated regions of greater structural flexibility [8,28].

#### 2.2.2. In Silico Cross-Reactivity Analysis

DNA aptamer candidates selected through an integrated assessment of comparative docking scores and molecular dynamics simulations were subjected to a final *in silico* cross-reactivity assessment before synthesis. Preliminary target selectivity was explored by molecular docking using AutoDock Vina, whereby each DNA aptamer candidate designed for one target protein was additionally assessed against the selected target-binding region of the non-target protein (e.g., IFI6-DNA aptamer candidates against the selected target-binding region of RSAD2 and vice versa). DNA aptamer candidates showing no predicted interactions within the selected target-binding regions of the non-target proteins were considered suitable for subsequent AHC assessment.

### 2.3. Aptahistochemistry Optimization

The purpose of the AHC experiments was to provide a preliminary assessment of the tissue reactivity of the *in silico* designed DNA aptamer candidates. Two archival FFPE OSCC tissue blocks previously confirmed to demonstrate positive IHC expression of IFI6 and RSAD2 using the corresponding primary antibodies (IFI6: PA5-99824; RSAD2: PA5-106337; Invitrogen, Waltham, MA, USA), together with healthy oral FFPE tissue blocks previously confirmed to demonstrate negative IHC expression of both proteins, were obtained from the Oral Pathology Laboratory, School of Dental Sciences, and the Pathology Laboratory, School of Medical Sciences, HUSM.

Following optimization, the selected IFI6- and RSAD2-DNA aptamer candidates were further evaluated using IFI6-positive kidney tissue and RSAD2-positive hepatocellular carcinoma tissue as positive control tissues to assess tissue reactivity. In addition, reciprocal tissue cross-reactivity assessment was performed by evaluating IFI6-targeting DNA aptamer candidates on RSAD2-positive hepatocellular carcinoma tissue and RSAD2-targeting DNA aptamer candidates on IFI6-positive kidney tissue, thereby providing a preliminary assessment of non-target tissue reactivity and target selectivity.

The selected *in silico* DNA aptamers were synthesized by Integrated DNA Technologies, Singapore, as biotinylated DNA aptamers and supplied in lyophilized form, and were subsequently used for optimization of AHC. Two antigen retrieval buffer solutions were evaluated during optimization: (1) Tris–EDTA (TE) buffer (pH 9) and (2) citrate buffer (pH 6). Antigen retrieval was performed at 100 °C and 110 °C, followed by incubation of the DNA aptamer-treated tissue sections for either 2 h at room temperature or overnight (14 h) at 4 °C to determine the optimal staining conditions. Tissue sections (2.5 µm) were deparaffinized, rehydrated in xylene, and graded with alcohols. Following antigen retrieval, the cooled sections were washed twice with Tris-buffered saline containing Tween-20 (TBST) and incubated with peroxidase-blocking solution (S202386; Dako–Agilent Technologies, Glostrup, Denmark). Biotinylated DNA aptamers (1 µM) were heated at 95 °C for 5 min and allowed to cool to room temperature to facilitate secondary-structure formation before application to the tissue sections. The sections were incubated overnight at 4 °C with the IFI6- and RSAD2-DNA aptamer candidates. After washing twice with TBST, streptavidin-HRP (ab64269; Abcam, Cambridge, UK) was applied for 30 min, followed by substrate chromogen solution (K5007; Dako–Agilent Technologies, Glostrup, Denmark) for 3 min and counterstaining with hematoxylin. The sections were dehydrated, mounted, and examined under light microscopy. Cytoplasmic brown granular staining in tumor cells was considered indicative of reactive DNA aptamer binding, comparable to the corresponding immunohistochemical staining patterns. The stained carcinomatous area was identified at 10× magnification, occupying a field that contains cancer cells, excluding the necrotic area [29]. A threshold of >50% reactive tumor cells was adopted from previously published cancer studies and was explored as a qualitative criterion during the optimization phase [30,31,32]. The IHC staining patterns served as reference standards for comparison during optimization. The qualitative evaluation was performed by a single clinical pathologist through direct comparison with the corresponding immunohistochemical staining results to assess DNA aptamer tissue reactivity during optimization. The images were captured using a scanner Motic EasyScan (Motic China Group Co., Ltd., Xiamen, China) at 20× power magnification. Following optimization, one IFI6-targeting and one RSAD2-targeting DNA aptamer candidate exhibiting staining patterns comparable to the corresponding IHC reference tissues were selected for subsequent cross-reactivity assessment. The cross-reactivity of the *in silico* aptamers was further qualitatively evaluated by AHC to assess preliminary target selectivity in tissue sections, using positive control tissues, namely, kidney tissue for IFI6 and liver cancer tissue for RSAD2 protein expression. The reciprocal positive control tissues were included to qualitatively assess tissue reactivity of each DNA aptamer candidate in both target and non-target tissues under identical experimental conditions. Representative images were acquired at 20× magnification using a scanner (Motic EasyScan).

## 3. Results

### 3.1. Generation of In Silico DNA Aptamers for IFI6 and RSAD2

**Figure 1:**
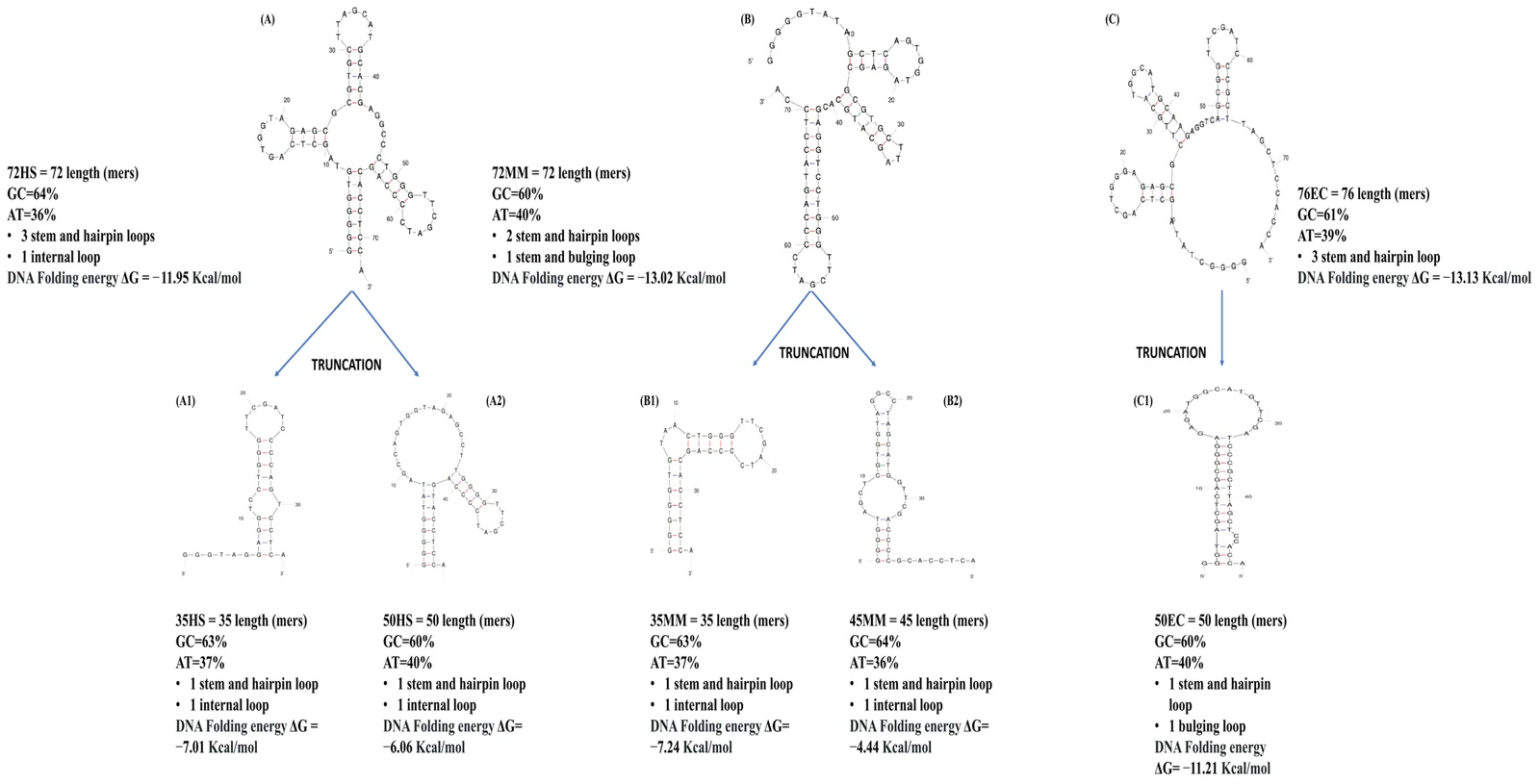
Secondary structure prediction and specification of truncated aptamer sequences. Primary sequences from *Homo sapiens*, *Mus musculus*, and *Escherichia coli* were truncated to generate five candidate DNA aptamer sequences (**A1**–**C1**). Panels (**A**–**C**) correspond to *Homo sapiens*, *Mus musculus*, and *Escherichia coli*–derived sequences, respectively, with predicted secondary structures shown for each truncated aptamer.

The data demonstrated that a reliable *in silico* DNA aptamer library containing primary sequences of varying mers can be constructed from different organisms using high isotype model fidelity and strong anticodon-scoring genomic sequences from GtRNAdb. By applying Chargaff’s second parity rule and sequence optimization principles during truncation, the method successfully generated multiple stem and hairpin loop 2D structures (Figure 1). These structures maintained nucleotide compositions (GC and AT content) within a ±5% range of the primary genomic sequences’ structural configurations (Table 1).

### 3.2. Molecular Docking and Molecular Dynamics Simulation of DNA Aptamers Interaction with IFI6 and RSAD2

**Figure 2:**
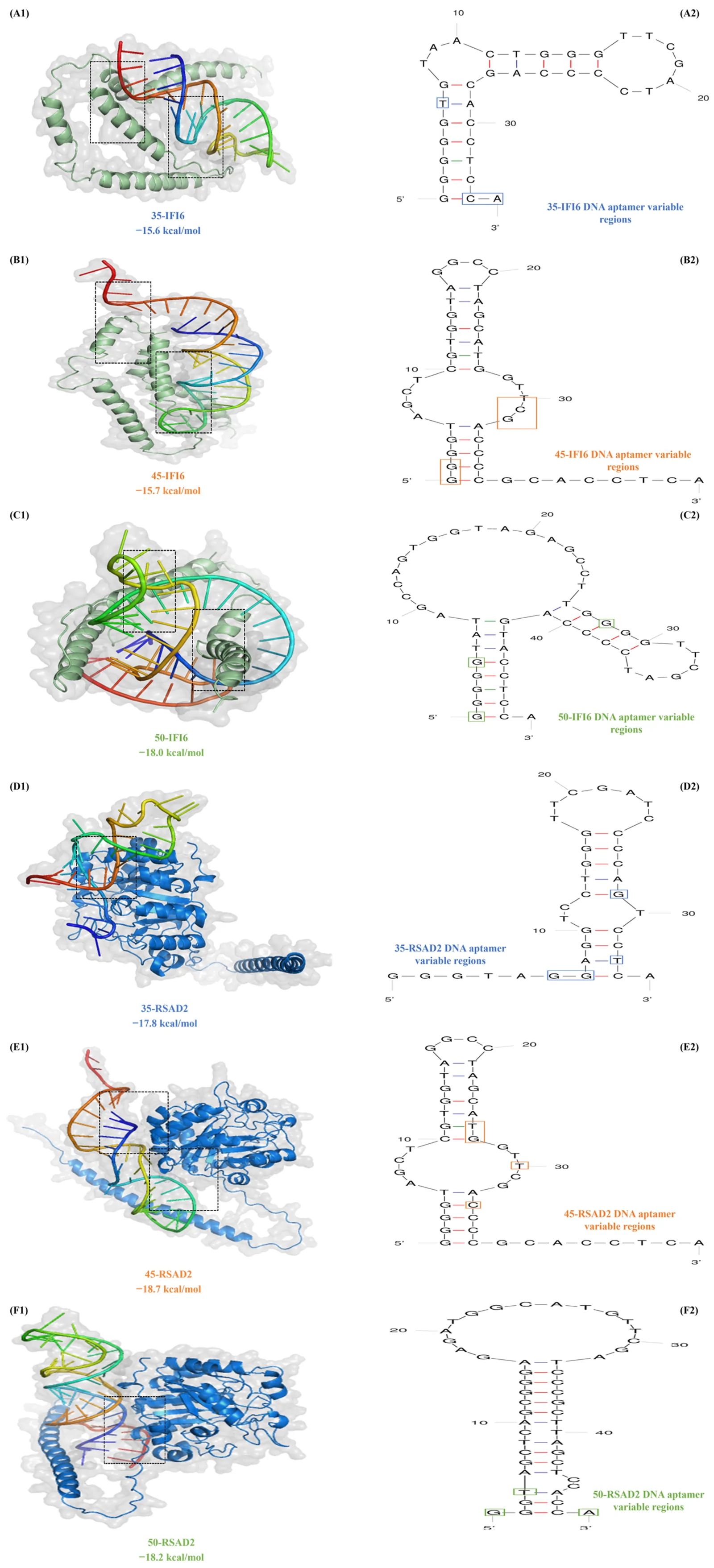
Comparative molecular docking analysis of IFI6- and RSAD2-targeting DNA aptamer candidates. Comparative docking scores and predicted interaction poses of the DNA aptamer candidates (**A1**–**F1**) with IFI6 and RSAD2 proteins are visualized using PyMOL. DNA aptamer candidates are displayed in cartoon representation with a rainbow color scheme, whereas IFI6 and RSAD2 proteins are shown in ribbon representation (IFI6 in green and RSAD2 in blue). The selected target-binding regions used for molecular docking are indicated by black dotted boxes (**A1**–**F1**). Corresponding secondary structure representations identify the interacting nucleotides within the IFI6-targeting DNA aptamer candidates (35-, 45-, and 50-IFI6), highlighted by blue, orange, and green boxes, respectively (**A2**–**C2**), and the RSAD2-targeting DNA aptamer candidates (35-, 45-, and 50-RSAD2), highlighted by blue, orange, and green boxes, respectively (**D2**–**F2**).

The selected IFI6 target-binding region (residues 1–50) corresponded to a comparatively low-confidence AlphaFold model (mean pLDDT = 52.90; range: 26.22–69.00), whereas the selected RSAD2 target-binding region (residues 170–361) corresponded to a high-confidence AlphaFold model (mean pLDDT = 95.31; range: 68.00–98.81). The DNA aptamer candidates exhibited favorable predicted interaction characteristics with the selected target-binding regions according to the comparative AutoDock Vina docking analysis (Figure 2; Table 2). Because the comparative docking scores differed within a relatively narrow range among the evaluated DNA aptamer candidates, molecular docking served primarily as an initial comparative screening approach before subsequent molecular dynamics simulations and exploratory tissue reactivity assessment. Among the IFI6-DNA aptamer candidates, 50-IFI6 exhibited the most favorable comparative docking score (−18.0 kcal/mol), followed by 45-IFI6 (−15.7 kcal/mol) and 35-IFI6 (−15.6 kcal/mol) (Figure 2(A1–C1)). Among the RSAD2-DNA aptamer candidates, 45-RSAD2 exhibited the most favorable comparative docking score (−18.7 kcal/mol), followed by 50-RSAD2 (−18.2 kcal/mol) and 35-RSAD2 (−17.8 kcal/mol) (Figure 2(D1–F1)). The interacting nucleotides were primarily located within the stem regions, with at least one interacting nucleotide contacting the target protein amino acid residues (Figure 2(A2–F2)). In the 45-IFI6, 35-RSAD2, and 45-RSAD2 DNA aptamer candidates, interacting nucleotides were located in both the stem and internal loop regions, suggesting that these structural regions may contribute to the predicted interaction profiles.

**Figure 3:**
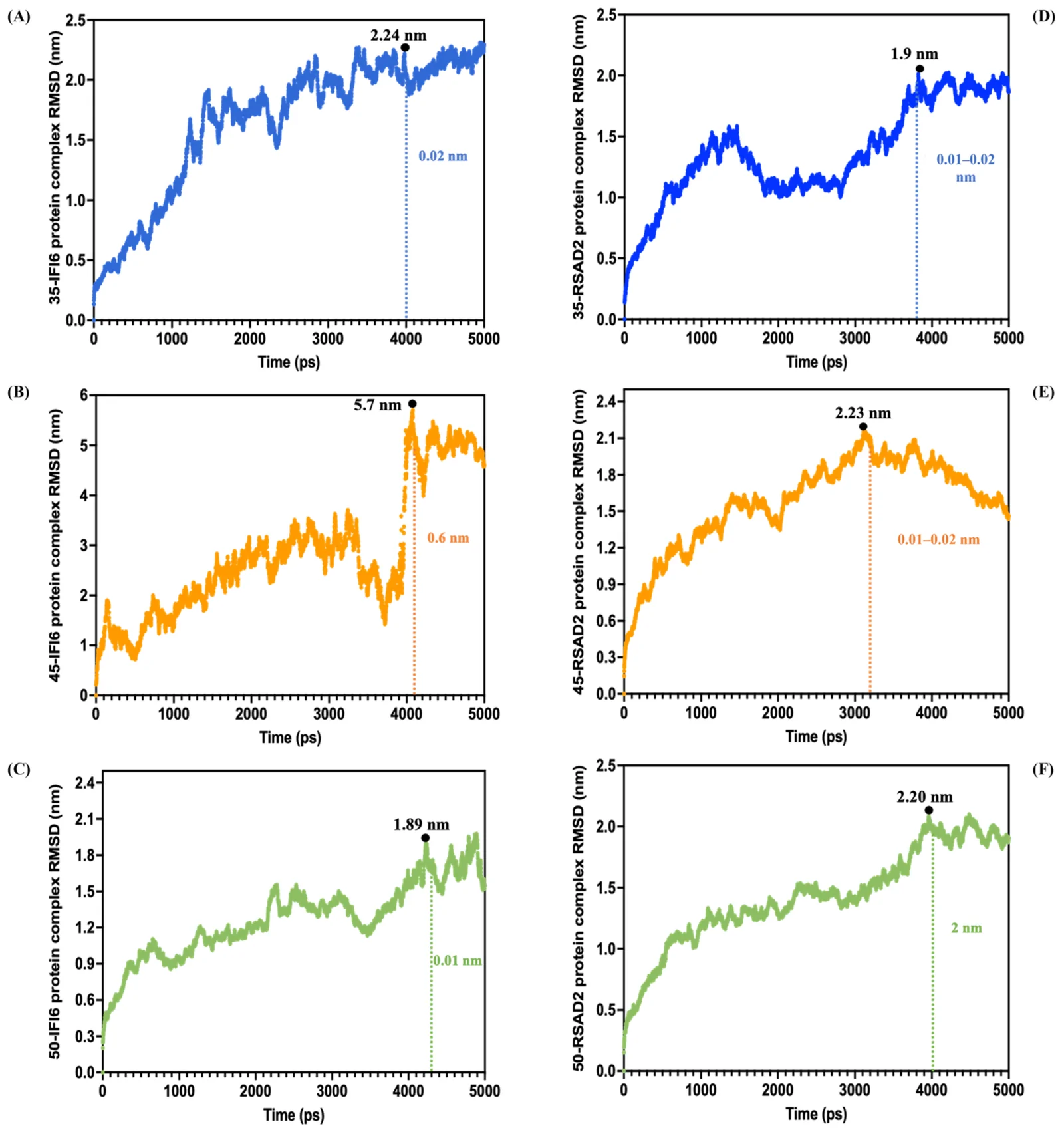
RMSD trajectories of IFI6–DNA aptamer complexes and RSAD2–DNA aptamer complexes during molecular dynamics simulations. IFI6–DNA aptamer complexes are shown in panels (**A**–**C**), and RSAD2–DNA aptamer complexes in panels (**D**–**F**), with blue, orange, and green curves representing the 35-, 45-, and 50-mer DNA aptamer candidates, respectively. Black dots indicate the maximum RMSD value observed for each complex, whereas the colored trajectories illustrate the structural deviations of the aptamer–protein complexes throughout the simulation, allowing comparative assessment of their structural behavior.

RMSD analysis was performed to evaluate the structural deviations of the aptamer–protein complexes throughout the molecular dynamics simulations. The 35-IFI6 DNA aptamer–protein complex exhibited a maximum RMSD of 2.24 nm at 3900 ps, after which the trajectory reached a plateau with relatively small fluctuations of 0.02 nm between 4000 and 5000 ps (Figure 3A). The 45-IFI6 DNA aptamer–protein complex reached a maximum RMSD of 5.70 nm at 4079 ps, after which the trajectory remained at comparatively high RMSD values with fluctuations of approximately 0.60 nm between 4100 and 5000 ps, without exhibiting a plateau characterized by relatively small fluctuations comparable to the other selected DNA aptamer complexes (Figure 3B). The 50-IFI6 DNA aptamer–protein complex exhibited a maximum RMSD of 1.89 nm at 4239 ps, after which the trajectory reached a plateau with fluctuations of 0.01 nm between 4300 and 5000 ps (Figure 3C). For the RSAD2 complexes, the 35-RSAD2 DNA aptamer–protein complex exhibited a maximum RMSD of 1.90 nm at 3740 ps, followed by a plateau with fluctuations of 0.01–0.02 nm between 3820 and 5000 ps (Figure 3D). Similarly, the 45-RSAD2 DNA aptamer–protein complex exhibited a maximum RMSD of 2.23 nm at 3100 ps, after which the trajectory reached a plateau with fluctuations of 0.01–0.02 nm between 3200 and 5000 ps (Figure 3E). In contrast, the 50-RSAD2 DNA aptamer–protein complex exhibited a maximum RMSD of 2.20 nm at 3940 ps, after which the trajectory remained close to 2.0 nm between 4000 and 5000 ps without exhibiting a comparable plateau characterized by small fluctuations (Figure 3F).

**Figure 4:**
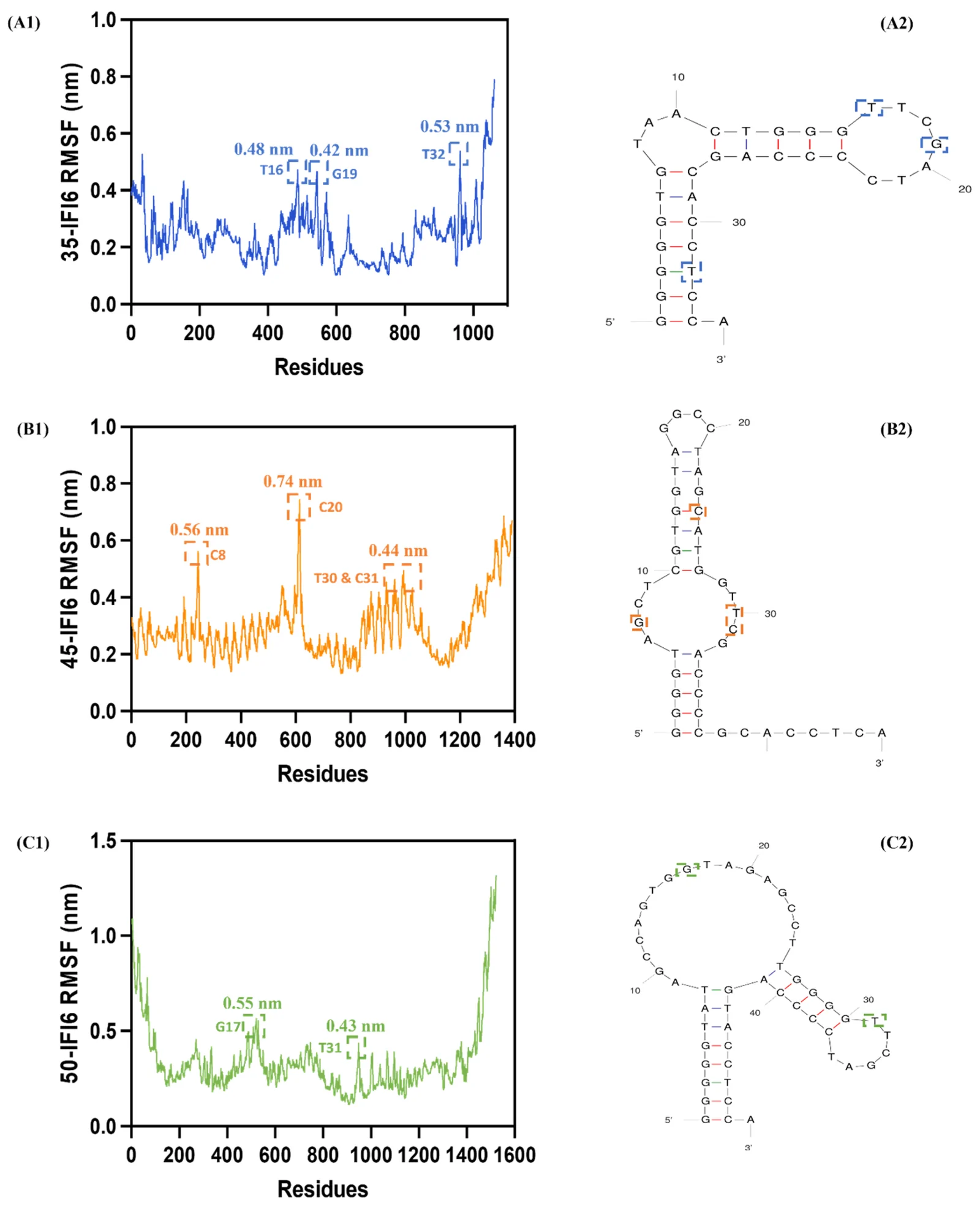
Root mean square fluctuation (RMSF) analysis and secondary structure region of IFI6-DNA aptamer candidates. RMSF plots generated using GraphPad Prism 9 illustrate positional RMSF values of IFI6-DNA aptamer candidates (35-, 45-, and 50-IFI6) during interaction with the IFI6 protein, with regions of increased flexibility highlighted by blue, orange, and green dotted boxes, respectively (**A1**–**C1**). Corresponding secondary structure representations of nucleotides in stem and hairpin loop regions, highlighted in blue, orange, and green font and enclosed within dotted boxes for the 35-, 45-, and 50-IFI6 DNA aptamers, respectively (**A2**–**C2**).

The reported atom indices correspond to the numbering in the GROMACS .gro coordinate files, whereas the nucleotide positions were assigned by mapping these atom indices to the corresponding nucleotides within each DNA aptamer sequence. RMSF analysis of the IFI6- and RSAD2-DNA aptamer candidates revealed varying degrees of local conformational flexibility within specific structural regions (Table 3). For the 35-IFI6 DNA aptamer, atom indices 480–487, 580, and 962 (corresponding to nucleotides T16, G19, and T32) exhibited RMSF values of 0.48, 0.42, and 0.53 nm, respectively, within the hairpin loop and stem regions. In the 45-IFI6 DNA aptamer, atom indices 240–247, 610–618, and 900–1000 (corresponding to nucleotides C8, C20, T30, and C31) exhibited RMSF values of 0.56, 0.74, and 0.44 nm, respectively, predominantly within the internal and hairpin loop regions. Likewise, atom indices 522–530 and 949 (corresponding to nucleotides G17 and T31) of the 50-IFI6 DNA aptamer exhibited RMSF values ranging from 0.43 to 0.55 nm within the hairpin loop region (Figure 4).

**Figure 5:**
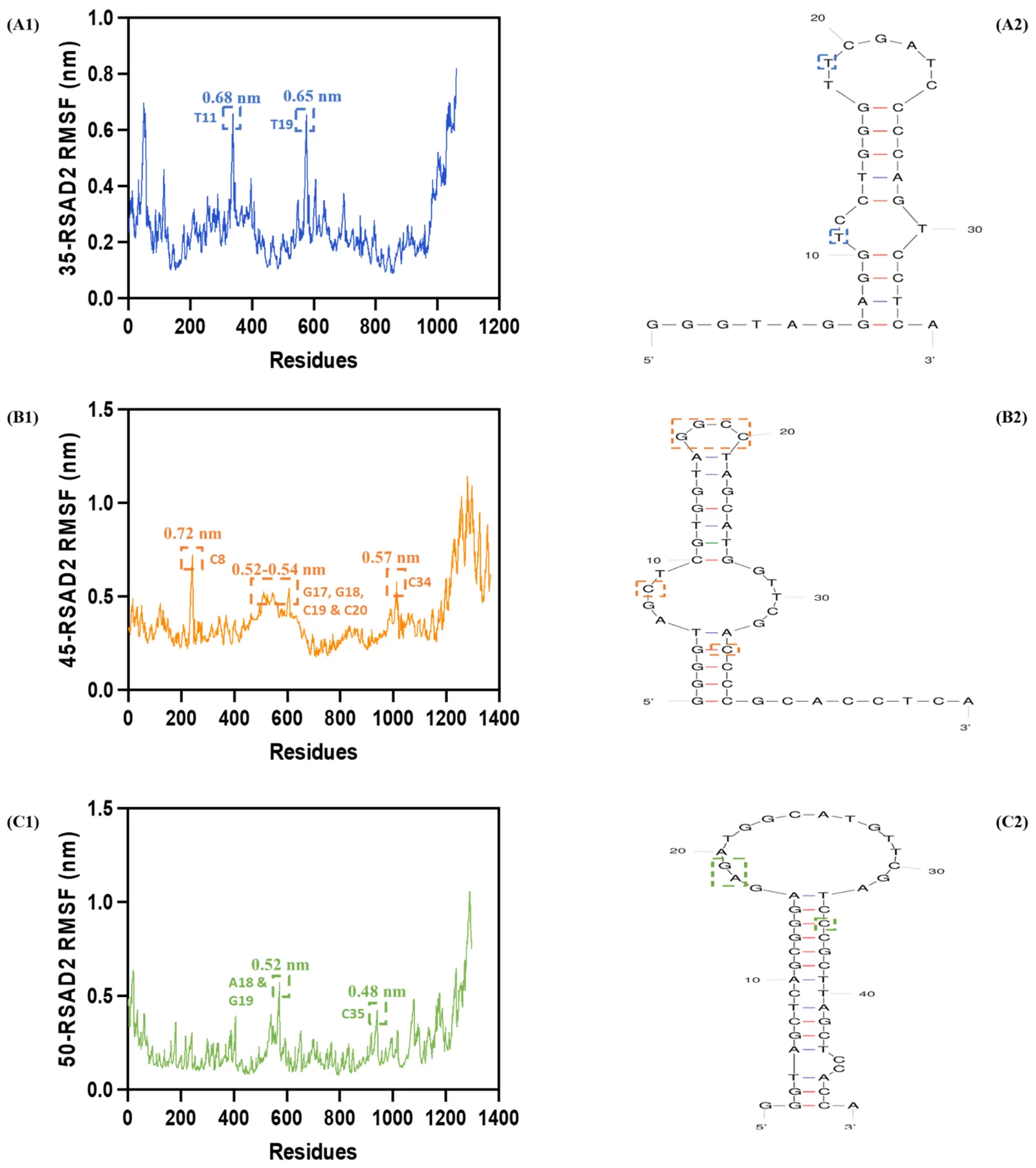
Root mean square fluctuation (RMSF) analysis and secondary structure region of RSAD2-DNA aptamer candidates. RMSF plots generated using GraphPad Prism 9 illustrate RMSF values of RSAD2-DNA aptamer candidates (35-, 45-, and 50-RSAD2) during interaction with the RSAD2 protein, with regions of increased flexibility highlighted by blue, orange, and green dotted boxes, respectively (**A1**–**C1**). Corresponding secondary structure representations of nucleotides in stem and hairpin loop regions, highlighted in blue, orange, and green font and enclosed within dotted boxes for the 35-, 45-, and 50-RSAD2 DNA aptamers, respectively (**A2**–**C2**).

For the 35-RSAD2 DNA aptamer, atom indices 334–341 and 570–579 (corresponding to nucleotides T11 and T19) exhibited RMSF values of 0.68 and 0.65 nm, respectively, within the internal and hairpin loop regions. In the 45-RSAD2 DNA aptamer, atom indices 233–245, 499–565, 600–610, and 1012 (corresponding to nucleotides C8, G17, G18, C19, C20, and C34) exhibited RMSF values of 0.72, 0.52, 0.54, and 0.57 nm, respectively, predominantly within the internal and hairpin loop regions. Similarly, atom indices 567–573 and 1076–1080 (corresponding to nucleotides A18, G19, and C35) of the 50-RSAD2 DNA aptamer exhibited RMSF values of 0.52 and 0.48 nm within the internal loop and stem regions, respectively (Figure 5).

### 3.3. Preliminary Assessment of In Silico Cross-Reactivity

**Figure 6:**
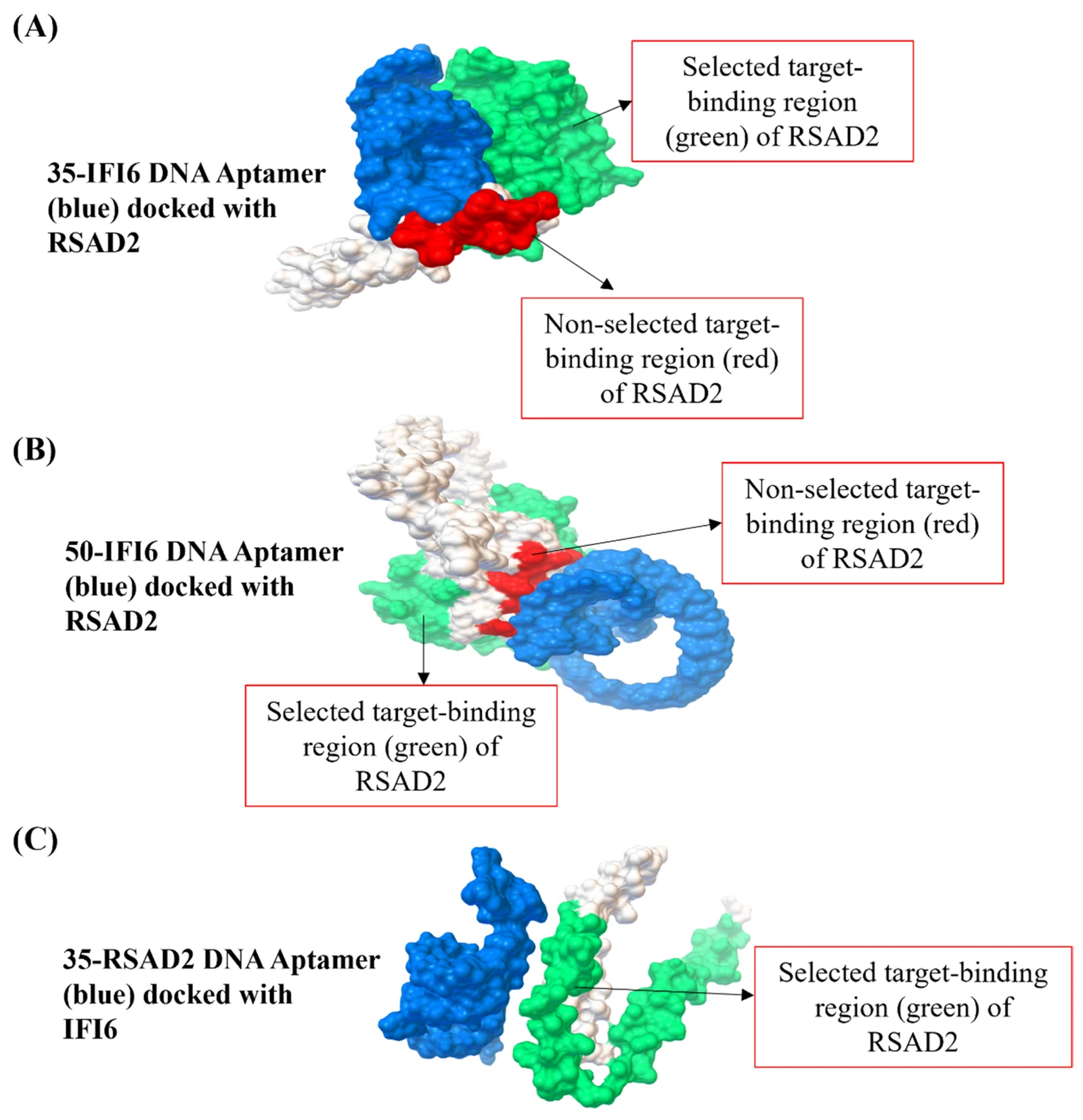
Preliminary *in silico* cross-reactivity assessment of IFI6- and RSAD2-targeting DNA aptamer candidates by molecular docking analysis. Molecular docking images generated using AutoDock Tools illustrate the preliminary cross-reactivity assessment between IFI6 and RSAD2 target proteins. Panels (**A**,**B**) show the 35-IFI6 and 50-IFI6 DNA aptamer candidates (blue molecular structures) interacting with amino acid residues outside the predefined target-binding region (green) of the RSAD2 protein (white molecular structure). Panel (**C**) shows that the 35-RSAD2 DNA aptamer candidate did not interact within the predefined target-binding region (green) of the IFI6 protein during the comparative molecular docking analysis.

The 45MM DNA aptamer candidate initially exhibited favorable comparative docking scores with both IFI6 and RSAD2. However, molecular dynamics simulations demonstrated that only the RSAD2 complex reached a plateau characterized by relatively small fluctuations following its maximum RMSD value, whereas the 45-IFI6 complex remained at comparatively higher RMSD values without exhibiting a comparable plateau. Consequently, the 45MM DNA aptamer candidate was prioritized as the RSAD2-targeting DNA aptamer candidate for subsequent cross-reactivity analysis, whereas the 45-IFI6 complex was excluded from further analyses. Therefore, the 45-RSAD2 DNA aptamer candidate was selected for subsequent AHC assessment. The remaining DNA aptamer candidates (35-IFI6, 50-IFI6, and 35-RSAD2) were selected following an integrated evaluation of comparative docking scores and molecular dynamics simulations for subsequent *in silico* cross-reactivity analysis (Figure 6). The 35-IFI6 DNA aptamer candidate interacted with amino acid residues LEU51, ARG54, and ASP57 outside the selected RSAD2 target-binding region, suggesting the absence of preferential interaction with the predefined RSAD2 target-binding region (Figure 6A). Similarly, the 50-IFI6 DNA aptamer candidate interacted with amino acid residues PHE98, LYS138, and ARG164 of RSAD2 outside the selected target-binding region (Figure 6B). Likewise, the 35-RSAD2 DNA aptamer candidate did not interact with the selected IFI6 target-binding region, supporting its preferential interaction with RSAD2 during the comparative computational screening process (Figure 6C).

### 3.4. Preliminary Tissue Reactivity Assessment

**Figure 7:**
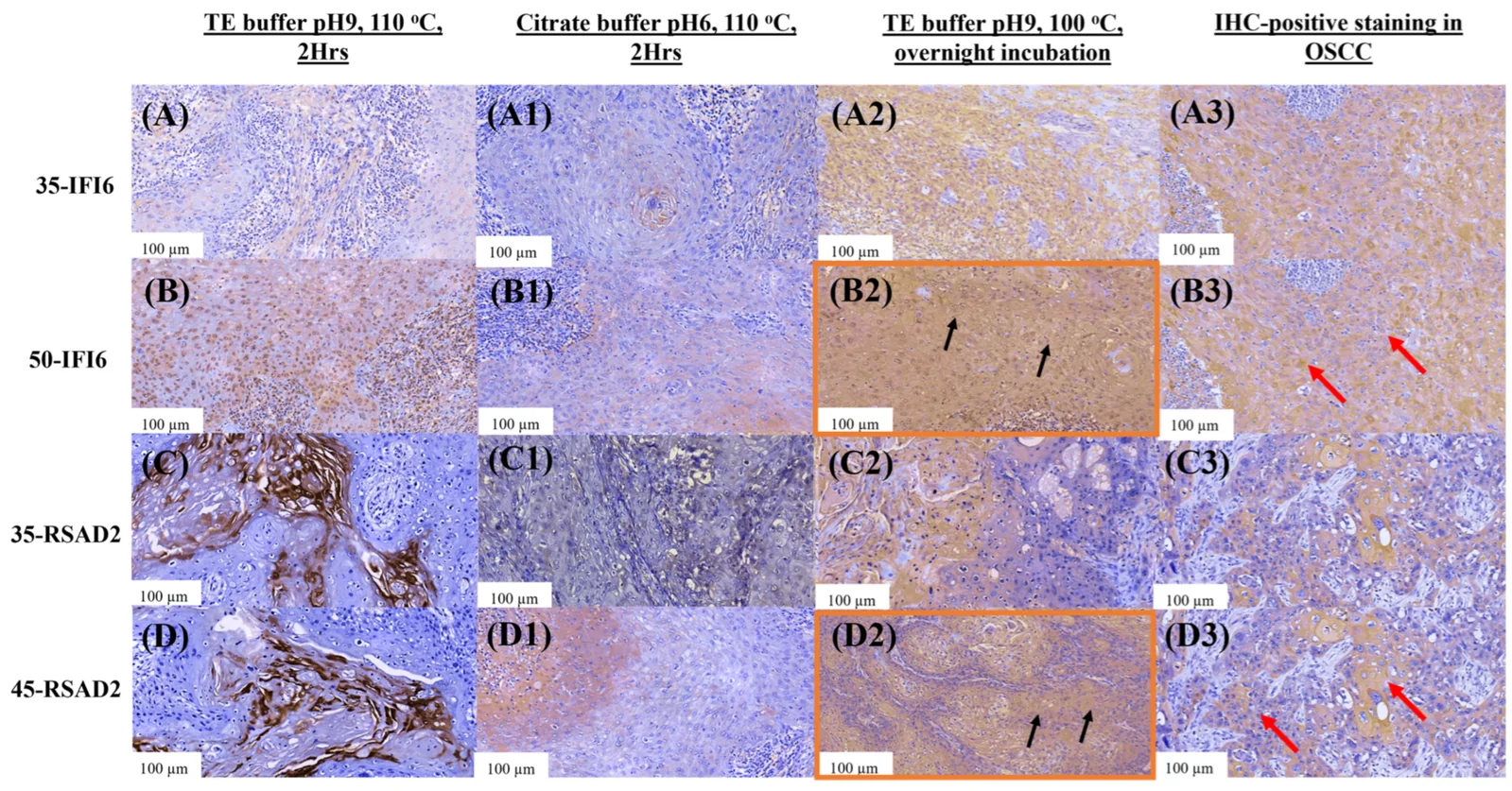
Optimization of *in silico* IFI6- and RSAD2-DNA aptamers by Aptahistochemistry in OSCC tissues. IFI6-targeting DNA aptamer candidates (35-IFI6 and 50-IFI6) and RSAD2-targeting DNA aptamer candidates (35-RSAD2 and 45-RSAD2) were assessed under different antigen retrieval buffer solutions, heat treatment temperatures, and incubation conditions. The experimental conditions included TE buffer (pH 9) at 110 °C followed by 2 h incubation at room temperature (**A**–**D**), citrate buffer (pH 6) at 110 °C followed by 2 h incubation at room temperature (**A1**–**D1**), and TE buffer (pH 9) at 100 °C followed by overnight incubation at 4 °C (**A2**–**D2**). The corresponding immunohistochemical (IHC) staining patterns for IFI6 and RSAD2 in OSCC tissues are shown in panels (**A3**–**D3**). Under the optimized experimental conditions, the 50-IFI6 (**B2**) and 45-RSAD2 (**D2**) DNA aptamer candidates exhibited cytoplasmic brown granular staining in >50% of tumor cells, as indicated by black arrows. Red arrows indicate the corresponding IHC staining patterns in OSCC tissues. Brown staining represents qualitative tissue reactivity of the DNA aptamer candidates. Scale bars = 100 µm. Images were acquired using a Motic Easy Scan scanner at 20× magnification.

The 35-IFI6, 50-IFI6, 35-RSAD2, and 45-RSAD2 DNA aptamer candidates were synthesized and qualitatively evaluated for preliminary tissue reactivity during AHC optimization. Variable staining intensities were observed under two antigen retrieval buffer solutions (TE buffer and citrate buffer), two heat treatment temperatures (100 °C and 110 °C), and two incubation conditions (2 h at room temperature and overnight at 4 °C) (Figure 7). Among the tested conditions, the 50-IFI6 and 45-RSAD2 DNA aptamer candidates exhibited the optimal tissue reactivity following antigen retrieval with TE buffer (pH 9) at 100 °C and overnight incubation at 4 °C. Under these optimized conditions, cytoplasmic brown granular staining was observed in >50% of tumor cells and was considered qualitatively reactive during the optimization process (Figure 7(B2,D2)). Distinct staining patterns were observed in OSCC tissues compared with the minimal or absent staining observed under the remaining optimization conditions. Representative cytoplasmic staining patterns comparable to the corresponding IHC reference tissues are shown in Figure 7.

### 3.5. Preliminary Tissue Cross-Reactivity Assessment in Positive Control Tissues

**Figure 8:**
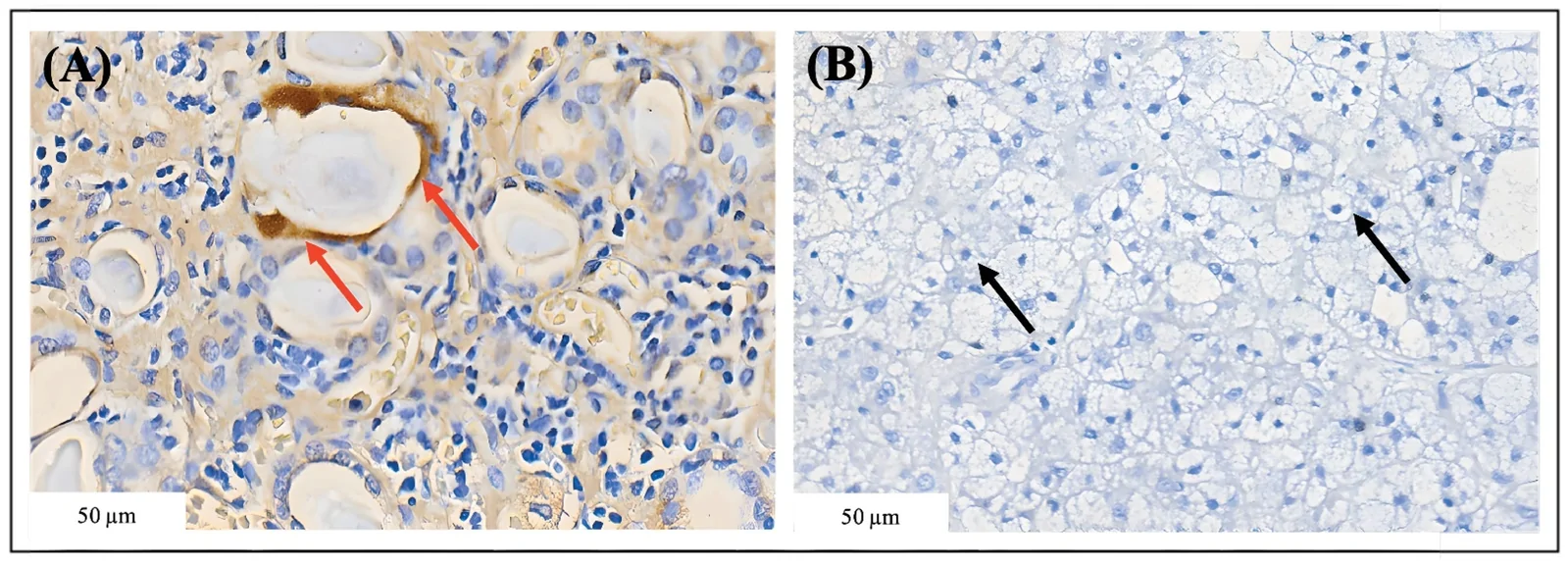
Preliminary cross-reactivity assessment of the IFI6-targeting DNA aptamer candidate in positive control tissues. Representative photomicrographs showing the 50-IFI6 DNA aptamer candidate at 40× magnification (scale bar = 50 µm) acquired using a Motic EasyScan scanner. (**A**) IFI6-positive control kidney tissue showing cytoplasmic staining in renal tubular epithelial cells (red arrows). (**B**) RSAD2-positive control hepatocellular carcinoma tissue showing the absence of cytoplasmic staining (black arrows). These observations qualitatively explored the tissue reactivity of the 50-IFI6 DNA aptamer candidate in target and non-target positive control tissues under identical experimental conditions.

**Figure 9:**
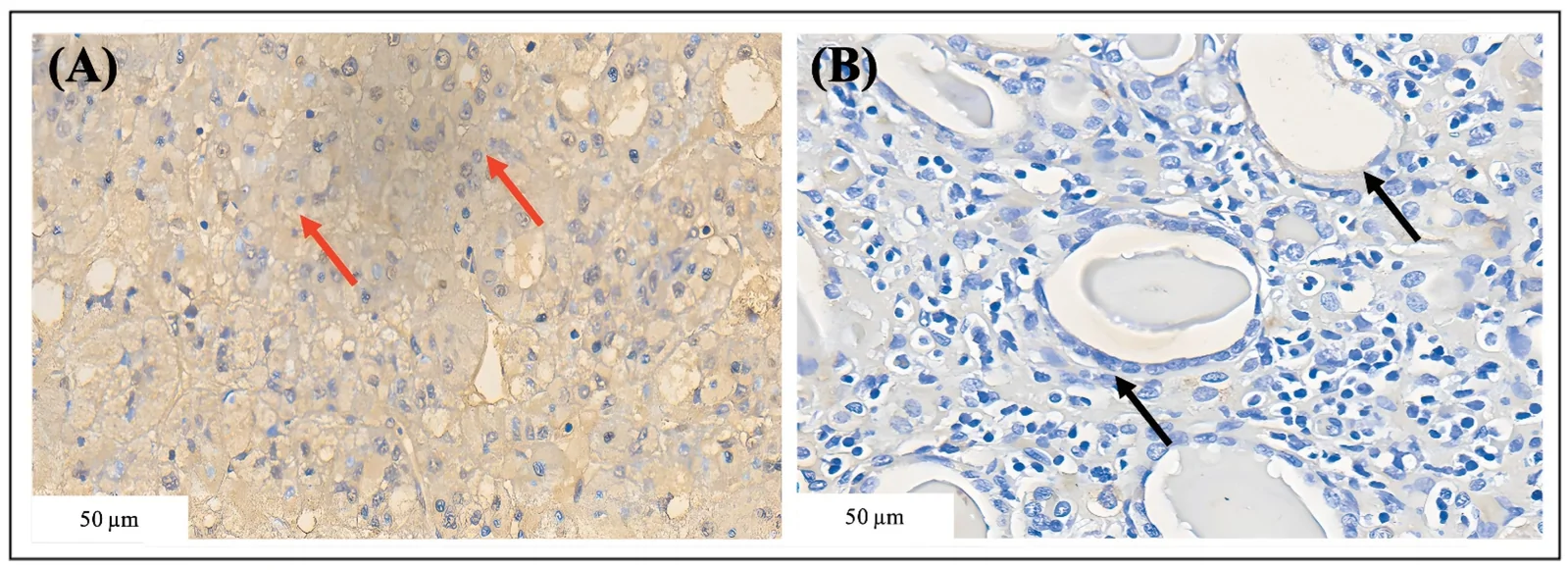
Preliminary cross-reactivity assessment of the RSAD2-targeting DNA aptamer candidate in positive control tissues. Representative photomicrographs showing the 45-RSAD2 DNA aptamer candidate at 40× magnification (scale bar = 50 µm) acquired using a Motic EasyScan scanner. (**A**) RSAD2-positive control kidney tissue showing cytoplasmic staining in hepatocellular carcinoma cells (red arrows). (**B**) IFI6-positive control kidney tissue showing the absence of cytoplasmic staining (black arrows). These observations qualitatively explored the tissue reactivity of the 50-IFI6 DNA aptamer candidate in target and non-target positive control tissues under identical experimental conditions.

The AHC assessment was performed to explore the preliminary target selectivity of the designed DNA aptamer candidates through qualitative comparison of staining patterns in target and non-target control tissues. The 50-IFI6 DNA aptamer candidate exhibited cytoplasmic staining in the renal tubular epithelial cells of kidney tissue, which served as the positive control for IFI6 expression, whereas no staining was observed in hepatocellular carcinoma tissue, which served as the positive control for RSAD2 expression (Figure 8). Conversely, the 45-RSAD2 DNA aptamer candidate exhibited cytoplasmic staining in hepatocellular carcinoma cells, the positive control for RSAD2 expression, whereas no staining was observed in kidney tissue, the positive control for IFI6 expression (Figure 9).

## 4. Discussion

The present study introduces a novel *in silico* design and characterization of DNA aptamers suitable for tissue-based protein detection. IFI6- and RSAD2-targeting DNA aptamers were computationally designed and analyzed before synthesis, addressing an important limitation in current aptamer development strategies for histopathological applications. Primary sequence acquisition was guided by high-confidence tRNA scores (>80), ensuring codon–anticodon fidelity and sequence reliability, as previously reported for biologically relevant nucleic acid modelling [33]. Secondary structure optimization involved systematic truncation while maintaining a balanced GC/AT composition, consistent with the general compositional principles described by Chargaff’s second parity rule, together with systematic removal of redundant loops and unpaired bases to preserve stable stem–loop and hairpin conformations [34,35,36], while extending them through a reproducible and structured optimization workflow. To the best of our knowledge, no previous study has reported a similarly integrated *in silico* strategy combining sequence scoring, structural truncation, molecular docking, molecular dynamics simulation, and preliminary tissue-based reactivity for the development of DNA aptamers intended for histopathological staining applications.

Importantly, the genomic tRNA-derived sequences used in the present study were not assumed to possess intrinsic affinity toward IFI6 or RSAD2. Instead, they served as structurally stable starting sequences that were subjected to systematic sequence truncation to generate DNA aptamer candidates for subsequent computational and experimental exploration. Rather than presuming target affinity from the parental genomic tRNA sequences, candidate prioritization was based on the combined assessment of molecular docking, molecular dynamics simulations, cross-reactivity assessment, and qualitative assessment of tissue reactivity.

Molecular docking scores generated by AutoDock Vina were interpreted as comparative ranking values for aptamer–protein interaction poses rather than as absolute thermodynamic binding free energies [37,38]. Accordingly, molecular docking was used primarily as a comparative screening approach to prioritize DNA aptamer candidates for subsequent molecular dynamics simulations, *in silico* cross-reactivity assessment, and tissue-based reactivity. Therefore, the interpretation of docking scores was restricted to relative ranking among DNA aptamer candidates evaluated under identical computational conditions within the present study, and direct thermodynamic comparisons with previously reported aptamer systems employing different targets, aptamer lengths, or computational protocols were avoided.

It is also acknowledged that AutoDock Vina was originally parameterized and validated for protein–small-molecule docking rather than large, structurally flexible nucleic acid ligands. Consequently, molecular docking in the present study was not intended to establish quantitative binding affinities but to provide a standardized computational framework for comparative candidate prioritization. Because the comparative docking scores among the evaluated DNA aptamer candidates differed within a relatively narrow range, molecular docking alone provided limited discriminatory power for candidate prioritization. The final selection of DNA aptamer candidates was therefore based on an integrated workflow combining molecular docking and molecular dynamics simulations, *in silico* cross-reactivity assessment, and subsequent aptahistochemical reactivity, rather than on molecular docking scores alone. Although redocking validation using experimentally characterized protein–nucleic acid complexes and specialized protein–nucleic acid docking algorithms may further strengthen future investigations, such analyses were beyond the scope of the present proof-of-concept study.

Previous studies have reported favorable aptamer–target comparative docking scores across diverse computational platforms [39]. The IFI6- and RSAD2-targeting DNA aptamer candidates yielded comparative docking scores ranging from −15.6 to −18.7 kcal/mol. Given the relatively narrow distribution of these scores, they were interpreted only as preliminary comparative ranking values generated under identical computational conditions rather than as quantitative indicators of binding strength. Consequently, molecular dynamics simulations, *in silico* cross-reactivity assessment, and subsequent aptahistochemical tissue reactivity were incorporated as complementary approaches for final candidate prioritization.

The comparatively lower AlphaFold confidence of the selected IFI6 docking region relative to the RSAD2 docking region should be considered when interpreting the molecular docking results. The docking findings, particularly for IFI6, should be interpreted as preliminary computational predictions within the context of this proof-of-concept investigation rather than definitive evidence of aptamer–protein interactions.

Molecular dynamics simulations performed using GROMACS were used to evaluate the structural behavior of the aptamer–protein complexes through RMSD analysis, consistent with previous studies investigating conformational changes during aptamer–target interactions [40,41]. Four DNA aptamer candidates (35-IFI6, 50-IFI6, 35-RSAD2, and 45-RSAD2) with their respective protein complexes exhibited RMSD values that reached a plateau with relatively small fluctuations during the latter stages of the simulation. In contrast, the remaining DNA aptamer candidates displayed comparatively larger fluctuations throughout the simulation period. These observations indicate differences in the structural behavior of the aptamer–protein complexes under the simulated conditions and provide an additional comparative criterion for candidate prioritization alongside molecular docking and cross-reactivity analyses.

The molecular dynamics simulations performed in the present study were intended to provide a preliminary assessment of the structural behavior of the aptamer–protein complexes during the 5000 ps (5 ns) simulation period. Accordingly, RMSD trajectories were interpreted as indicators of structural deviations relative to the initial docked complex rather than as definitive evidence of long-term binding stability or continuous retention of the aptamers within the predicted target-binding regions. Because contact occupancy, distance analyses, and binding-pocket retention analyses were not performed, conclusions regarding sustained target engagement were intentionally limited. Therefore, the molecular dynamics results should be interpreted as a comparative assessment of the structural behavior of the aptamer–protein complexes under the simulated conditions rather than as definitive evidence of long-term binding stability.

The newer nucleic acid force fields, such as AMBER bsc1 and OL15, have been developed to improve the representation of long-timescale nucleic acid dynamics [42]. The present study employed AMBER94 because all DNA aptamer candidates were evaluated under identical simulation conditions for comparative computational assessment. Consequently, the relative structural comparisons among DNA aptamer candidates remained internally consistent within the present study. Nevertheless, the use of AMBER94, a single 5 ns production simulation, and the absence of replicate simulations represent limitations of this proof-of-concept investigation. Accordingly, the molecular dynamics results should be interpreted as a comparative assessment of the structural behavior of the DNA aptamer–protein complexes under the simulated conditions rather than as definitive evidence of long-term binding stability. Future studies employing next-generation nucleic acid force fields, longer simulation times, replicate simulations, and additional contact occupancy or distance analyses may provide a more comprehensive evaluation of the conformational behavior and interaction characteristics of the developed DNA aptamer candidates.

The converted DNA aptamers from RNA in PyMol served as the initial templates for molecular docking. The resulting docked complexes were subsequently subjected to steepest descent energy minimization, equilibration, and molecular dynamics simulations, allowing structural relaxation under the applied simulation conditions [43,44].

Structural optimization of the stem and hairpin loop regions further contributed to the semi-flexible properties of the DNA aptamers. Semi-flexibility was achieved by retaining rotatable phosphate–oxygen bonds while restricting carbon-based bond rotations within AutoDock Tools, thereby balancing conformational adaptability with structural rigidity. This approach is supported by previous studies demonstrating the importance of flexible loop regions in facilitating aptamer interaction with glucose, N-acetylneuraminic acid, and carcinoembryonic antigen [36,45]. RMSF values in the current study demonstrated increased flexibility within loop regions (>0.4 nm), whereas stem regions exhibited comparatively lower RMSF values, indicating distinct patterns of local conformational flexibility within the DNA aptamer candidates during the molecular dynamics simulations. These observations are consistent with previous reports describing flexible “coil” regions as important determinants of aptamer binding efficiency [46,47,48]. Interestingly, the 45MM DNA aptamer candidate exhibited variable flexibility when evaluated with IFI6 and RSAD2, suggesting target-dependent conformational adaptation during the molecular dynamics simulations.

In this proof-of-concept investigation, the AHC findings should be interpreted as an exploratory, preliminary assessment of tissue reactivity obtained during optimization of the developed DNA aptamer candidates rather than as independent evidence of analytical specificity or clinical diagnostic performance. Among the tested optimization conditions, antigen retrieval with TE buffer (pH 9) at 100 °C followed by overnight incubation at 4 °C produced the most consistent tissue reactivity for the 50-IFI6 and 45-RSAD2 DNA aptamer candidates, characterized by cytoplasmic granular staining in >50% of tumor cells comparable to the corresponding IHC staining patterns. These findings suggest that the alkaline TE buffer (pH 9), together with prolonged incubation, may have facilitated aptamer–target interactions by improving epitope accessibility within formalin-fixed paraffin-embedded tissues. Previous studies have similarly demonstrated that aptamer-based histochemistry is highly dependent on optimization of buffer composition, temperature, and incubation parameters to preserve aptamer conformational integrity and maximize target-specific binding [19]. In particular, alkaline buffer systems have been reported to promote stable secondary structure formation and improve hybridisation efficiency of nucleic acid probes in tissue environments. Furthermore, prolonged incubation may facilitate gradual structural adaptation of the aptamer towards exposed antigenic regions, thereby enhancing preferential target binding and signal intensity. Therefore, these findings suggest that optimization of antigen retrieval buffer solutions and incubation conditions may be important for achieving staining patterns comparable to conventional immunohistochemistry.

The observed concordance between the computational predictions and the exploratory AHC tissue reactivity assessment supports the applicability of the proposed *in silico* workflow for DNA aptamer candidate development. The qualitative tissue assessment was performed by a single pathologist using a predefined criterion of >50% reactive tumor cells during optimization. The optimization experiments intentionally employed immunohistochemically characterized FFPE tissues as reference tissues to optimize aptahistochemical conditions and qualitatively evaluate tissue reactivity of the selected DNA aptamer candidates under different experimental conditions, analogous to optimization procedures routinely employed in antibody-based tissue detection methods. Therefore, the observed similarity between AHC and the corresponding IHC staining patterns should be interpreted as the outcome of optimization using established reference tissues rather than as an independent assessment of analytical specificity. Although healthy oral tissues and reciprocal positive control tissues were incorporated as biological controls during the optimization and cross-reactivity assessments, these controls do not substitute for analytical specificity controls. Accordingly, future studies incorporating scrambled-sequence aptamers, no-aptamer controls, competition assays, quantitative binding analyses (e.g., surface plasmon resonance [SPR] and microscale thermophoresis [MST]), pull-down assays, cellular internalization studies, functional assays, quantitative image analyses, blinded assessments, inter-observer evaluations, dual staining approaches for protein co-localization, and larger validation cohorts will be important to establish reproducibility, analytical specificity, target selectivity, and broader translational applicability of the developed IFI6- and RSAD2-DNA aptamer candidates.

Preliminary target selectivity of the designed DNA aptamer candidates was explored through integrated *in silico* cross-reactivity analysis and tissue-based AHC assessment. Molecular docking indicated that the IFI6-DNA aptamer candidates did not interact with the selected target-binding region of RSAD2, whereas the RSAD2-DNA aptamer candidates similarly showed no interactions within the selected target-binding region of IFI6. These findings provided preliminary computational evidence suggesting reduced off-target interactions before tissue application. The computational observations were subsequently explored through qualitative AHC assessment, where each DNA aptamer candidate exhibited cytoplasmic staining in its corresponding positive control tissue while showing no staining in the reciprocal non-target control tissue. Specifically, the IFI6-DNA aptamer candidate exhibited cytoplasmic staining in renal tubular epithelial cells without staining in hepatocellular carcinoma tissue, whereas the RSAD2-DNA aptamer candidate exhibited cytoplasmic staining in hepatocellular carcinoma tissue without staining in kidney tissue. Collectively, these findings support the exploratory application of the integrated *in silico* workflow for identifying DNA aptamer candidates with preliminary target selectivity; however, additional analytical specificity studies incorporating appropriate negative controls and quantitative binding assays are required to confirm these observations.

## 5. Conclusions

The integration of computational modelling, molecular docking, molecular dynamics simulations, cross-reactivity analysis, and exploratory aptahistochemical assessment supports the applicability of an integrated *in silico* DNA aptamer development workflow for generating structurally optimized DNA aptamer candidates with preliminary tissue reactivity. Furthermore, the exploratory tissue reactivity observed for the IFI6- and RSAD2-DNA aptamer candidates supports continued investigation of their biological relevance. Collectively, these findings provide preliminary proof-of-concept for the application of computationally engineered DNA aptamer candidates in histopathological research and warrant further analytical, biological, and clinical validation studies incorporating expanded cohorts, comprehensive target selectivity assessment, appropriate analytical specificity controls, and quantitative binding analyses.

## Figures and Tables

**Table 1:** Specifications of truncated sequences.

Primary Sequence -mers	Primary Sequence	Truncated Aptamer Candidate -mers	Truncated Aptamer Sequence	GC (%)	AT (%)
72HS	5′-GGGGGTGTAGCTCAGTGGTA GAGCGCGTGC TTAGCATGCACGAGGCCCTGGGTTCGATCCCCAGCACCTC CA-3′	35	5′-GGGTAGGAGG TCCTGGGTTC GATCCCCAGT CCTCA-3′	22 (63%)	13 (37%)
		50	5′-GGGGGTATAG CCAGTGGTAG AGCCTTGGGG TTCGATCCCC AGTACCTCCA-3′	30 (60%)	20 (40%)
72MM	5′-GGGGGTATAGCTCAGTGGTAGAGCGCGTGCTTAGCATGCACGAGGTCCTGGGTTCGATCCCCAGTACCTC CA-3′	35	5′-GGGGGTGTAA CTGGGTTCGA TCCCCAGCAC CTCCA-3′	22 (63%)	13 (37%)
		45	5′-GGGGTAGCTC GTGGTAGGCC TAGCATGGTT CGACCCCGCA CCTCA-3′	29 (64%)	16 (36%)
76EC	5′-GGGGCTATAGCTCAGCTGGGAGAGCGCTTG CATGGCATGCAAGAGGTCAGCGGTTCGATC CCGCTTAGCTCCACCA-3′	50	5′-GGGTAGCTCA GCGGGAGAGA TGGCATGTTC GATCCCGCTT AGCTCCACCA-3′	30 (60%)	20 (40%)

Abbreviations: G (Guanine), C (Cytosine), T (Thymine), A (Adenine), GC (Guanine–Cytosine), AT (Adenine–Thymine).

**Table 2:** Binding interaction analysis of IFI6- and RSAD2-DNA aptamer candidates.

DNA Aptamer Candidates	Comparative Docking Scores (kcal/mol)	DNA Aptamer Nucleotides-Protein Amino Acid Residues
35-IFI6	−15.6	DG1-THR17 DT6-CYS18 DC34-VAL21 DC34-GLY24 DA35-GLY24
45-IFI6	−15.7	DG1-MET1 DG2-LEU8 DT30-SER7 DC31-LEU14 DC32-LEU15
50-IFI6	−18.0	DG1-THR17 DG5-PHE44 DG5-GLY48 DG28-CYS18
35-RSAD2	−17.8	DG6-LEU252 DG7-GLU267 DG29-ASP273 DT33-ASN225 DT33-ARG268
45-RSAD2	−18.7	DG4-ARG215 DT26-ASP356 DG28-GLU171 DG28-ARG215 DT30-GLU172
50-RSAD2	−18.2	DG1-ARG346 DG4-LYS219 DA50-TRP245

Abbreviations: DG (DNA-Guanine), DC (DNA-Cytosine), DT (DNA-Thymine), DA (DNA-Adenine), SER (Serine), THR (Threonine), CYS (Cystine), GLY (Glycine), LEU (Leucine), VAL (Valine), Methionine (MET), PHE (Phenylalanine), LYS (Lysine), GLU (Glutamic acid), ASP (Aspartic acid), ARG (Arginine), TRP (Tryptophan), ASN (Asparagine). Docking scores represent comparative ranking values generated under identical computational conditions and should not be interpreted as quantitative binding free energies.

**Table 3:** RMSF analysis of IFI6- and RSAD2-DNA aptamers.

DNA Aptamers	Atom Indices (.gro File)	Corresponding Nucleotides	RMSF (nm)	Structural Region
35-IFI6	480–487 580 962	T (16) G (19) T (32)	0.48 0.42 0.53	Hairpin loop Hairpin loop Stem
45-IFI6	240–247 610–618 900–1000	C (8) C (20) T (30) and C (31)	0.56 0.74 0.44	Internal loop Hairpin loop Internal loop
50-IFI6	522–530 949	G (17) T (31)	0.55 0.43	Hairpin loop Hairpin loop
35-RSAD2	334–341 570–579	T (11) T (19)	0.68 0.65	Internal loop Hairpin loop
45-RSAD2	233–245 499–565 600–610 1012	C (8) G (17), G (18), and C (19) C (20) C (34)	0.72 0.52 0.54 0.57	Internal loop Hairpin loop Hairpin loop Stem
50-RSAD2	567–573 1076–1080	A (18) and G (19) C (35)	0.52 0.48	Internal loop Stem

Abbreviations: G (Guanine), C (Cytosine), T (Thymine), A (Adenine), GC (Guanine–Cytosine), AT (Adenine–Thymine). Atom indices correspond to the numbering in the GROMACS .gro coordinate files and were mapped to the corresponding nucleotide(s) within each DNA aptamer sequence.

## Data Availability

The original contributions presented in this study are included in the article. Further inquiries can be directed to the corresponding authors.

## Abbreviations

The following abbreviations are used in this manuscript

A	Adenine
AHC	Aptahistochemistry
AT	Adenine–Thymine
C	Cytosine
DNA	Deoxyribonucleic Acid
EC	Escherichia coli
FFPE	Formalin-Fixed Paraffin-Embedded
G	Guanine
GC	Guanine–Cytosine
GROMACS	Groningen Machine for Chemical Simulations
GtRNAdb	Genomic Transfer RNA Database
HNSCC	Head and Neck Squamous Cell Carcinoma
HRP	Horseradish Peroxidase
HS	Homo sapiens
HUSM	Hospital Universiti Sains Malaysia
IDT	Integrated DNA Technologies
IFI6	Interferon Alpha-Inducible Protein 6
IHC	Immunohistochemistry
MD	Molecular Dynamics
MM	Mus musculus
mRNA	Messenger Ribonucleic Acid
OSCC	Oral Squamous Cell Carcinoma
PDB	Protein Data Bank
qVina2	Quick AutoDock Vina 2
RMSD	Root Mean Square Deviation
RMSF	Root Mean Square Fluctuation
RNA	Ribonucleic Acid
RSAD2	Radical S-Adenosyl Methionine Domain-Containing Protein 2
SELEX	Systematic Evolution of Ligands by Exponential Enrichment
ssDNA	Single-Stranded DNA
ssRNA	Single-Stranded RNA
T	Thymine
TBST	Tris-Buffered Saline Tween-20
TE	Tris–EDTA
tRNA	Transfer RNA
UniProt	Universal Protein Resource
USM	Universiti Sains Malaysia

## References

[1] Elmusrati A., Wang J., Wang C.Y. (2021). Tumor microenvironment and immune evasion in head and neck squamous cell carcinoma. Int. J. Oral Sci..

[2] Bray F., Laversanne M., Sung H., Ferlay J., Siegel R.L., Soerjomataram I., Jemal A. (2024). Global cancer statistics 2022: GLOBOCAN estimates of incidence and mortality worldwide for 36 cancers in 185 countries. CA Cancer J. Clin..

[3] Jeddi I., Saiz L. (2017). Three-dimensional modeling of single stranded DNA hairpins for aptamer-based biosensors. Sci. Rep..

[4] Nimjee S.M., White R.R., Becker R.C., Sullenger B.A. (2017). Aptamers as therapeutics. Annu. Rev. Pharmacol. Toxicol..

[5] Subramanian N., Akilandeswari B., Bhutra A., Alameen M., Vetrivel U., Khetan V., Kanwar R.K., Kanwar J.R., Krishnakumar S. (2016). Targeting CD44, ABCG2 and CD133 markers using aptamers: In silico analysis of CD133 extracellular domain 2 and its aptamer. RSC Adv..

[6] Ahirwar R., Nahar S., Aggarwal S., Ramachandran S., Maiti S., Nahar P. (2016). In silico selection of an aptamer to estrogen receptor alpha using computational docking employing estrogen response elements as aptamer-like molecules. Sci. Rep..

[7] Lopez-Silva C., Surapaneni A., Coresh J., Reiser J., Parikh C.R., Obeid W., Grams M.E., Chen T.K. (2022). Comparison of aptamer-based and antibody-based assays for protein quantification in chronic kidney disease. Clin. J. Am. Soc. Nephrol..

[8] Bavi R., Hang Z., Banerjee P., Aquib M., Jadhao M., Rane N., Bavi S., Bhosale R., Kodam K., Jeon B.-H. (2020). Doxorubicin-conjugated innovative 16-mer DNA aptamer-based annexin A1 targeted anti-cancer drug delivery. Mol. Ther. Nucleic Acids.

[9] Li L., Wan J., Wen X., Guo Q., Jiang H., Wang J., Ren Y., Wang K. (2021). Identification of a new DNA aptamer by tissue-SELEX for cancer recognition and imaging. Anal. Chem..

[10] Zhuo Z., Yu Y., Wang M., Li J., Zhang Z., Liu J., Wu X., Lu A., Zhang G., Zhang B. (2017). Recent advances in SELEX technology and aptamer applications in biomedicine. Int. J. Mol. Sci..

[11] Soon S., Nordin N.A. (2019). In silico predictions and optimization of aptamers against Streptococcus agalactiae surface protein using computational docking. Mater. Today Proc..

[12] Qi Y., Li Y., Zhang Y., Zhang L., Wang Z., Zhang X., Gui L., Huang J. (2015). IFI6 inhibits apoptosis via mitochondrial-dependent pathway in dengue virus 2 infected vascular endothelial cells. PLoS ONE.

[13] Li Y., Cui Q., Zhou B., Zhang J., Guo R., Wang Y., Xu X. (2024). RSAD2, a pyroptosis-related gene, predicts the prognosis and immunotherapy response for colorectal cancer. Am. J. Cancer Res..

[14] Huang C., Zhou T., Ma L., Zhao S. (2023). IFI6 downregulation reverses oxaliplatin resistance of colorectal cancer cells by activating the ROS-induced p38MAPK signaling pathway. Biol. Pharm. Bull..

[15] Xu L., Zu T., Li T., Li M., Mi J., Bai F., Liu G., Wen J., Li H., Brakebusch C. (2021). ATF3 downmodulates its new targets IFI6 and IFI27 to suppress the growth and migration of tongue squamous cell carcinoma cells. PLoS Genet..

[16] Choi K.M., Kim J.J., Yoo J., Kim K.S., Gu Y., Eom J., Jeong H., Kim K., Nam K.T., Park Y.S. (2022). The interferon-inducible protein viperin controls cancer metabolic reprogramming to enhance cancer progression. J. Clin. Investig..

[17] Singh P., Rai A., Verma A.K., Alsahli M.A., Rahmani A.H., Almatroodi S.A., Alrumaihi F., Dev K., Sinha A., Sankhwar S. (2021). Survival-based biomarker module identification associated with oral squamous cell carcinoma (OSCC). Biology.

[18] Bergado-Báez G., Suarez N.G., García L.C., Pérez-Martínez D., Hernández-Fernández D.R., Fundora-Barrios T., Rodríguez-Álvarez A., Díaz-Ordaz G.D., Lindzen M., Yarden Y. (2022). Polyclonal antibody-induced downregulation of HER1/EGFR and HER2 surpasses the effect of combinations of specific registered antibodies. Front. Oncol..

[19] Liu M., Xi L., Tan T., Jin L., Wang Z., He N. (2021). A novel aptamer-based histochemistry assay for specific diagnosis of clinical breast cancer tissues. Chin. Chem. Lett..

[20] Butt D.Q. (2025). Elucidating IFI6 and RSAD2 Protein Expression in Oral Squamous Cell Carcinoma Using Immunohistochemistry and Aptahistochemistry with Novel In Silico DNA Aptamers. Ph.D. Thesis.

[21] Chan P.P., Lowe T.M. (2016). GtRNAdb 2.0: An expanded database of transfer RNA genes identified in complete and draft genomes. Nucleic Acids Res..

[22] Rudner R., Karkas J.D., Chargaff E. (1968). Separation of *B. subtilis* DNA into complementary strands. III. Direct analysis. Proc. Natl. Acad. Sci. USA.

[23] Wan L., Yoshikawa A., Eisenstein M., Soh H.T. (2023). High-throughput strategy for enhancing aptamer performance across different environmental conditions. ACS Sens..

[24] Popenda M., Szachniuk M., Antczak M., Purzycka K.J., Lukasiak P., Bartol N., Blazewicz J., Adamiak R.W. (2012). Automated 3D structure composition for large RNAs. Nucleic Acids Res..

[25] Chen Y., Zhang H., Wang W., Shen Y., Ping Z. (2024). Rapid generation of high-quality structure figures for publication with PyMOL-PUB. Bioinformatics.

[26] Morris G.M., Huey R., Lindstrom W., Sanner M.F., Belew R.K., Goodsell D.S., Olson A.J. (2009). AutoDock4 and AutoDockTools4: Automated docking with selective receptor flexibility. J. Comput. Chem..

[27] Trott O., Olson A.J. (2010). AutoDock Vina: Improving the speed and accuracy of docking with a new scoring function, efficient optimization, and multithreading. J. Comput. Chem..

[28] Abraham M.J., Murtola T., Schulz R., Páll S., Smith J.C., Hess B., Lindahl E. (2015). GROMACS: High performance molecular simulations through multi-level parallelism from laptops to supercomputers. SoftwareX.

[29] Balermpas P., Rödel F., Rödel C., Krause M., Linge A., Lohaus F., Baumann M., Tinhofer I., Budach V., Gkika E. (2016). CD8+ tumour-infiltrating lymphocytes in relation to HPV status and clinical outcome in patients with head and neck cancer after postoperative chemoradiotherapy: A multicentre study of the German cancer consortium radiation oncology group (DKTK-ROG). Int. J. Cancer.

[30] Bauer M., Macdonald J., Henri J., Duan W., Shigdar S. (2016). The application of aptamers for immunohistochemistry. Nucleic Acid Ther..

[31] McCampbell A.S., Raghunathan V., Tom-Moy M., Workman R.K., Haven R., Ben-Dor A., Rasmussen O.F., Jacobsen L., Lindberg M., Yamada N.A. (2019). Tissue thickness effects on immunohistochemical staining intensity of markers of cancer. Appl. Immunohistochem. Mol. Morphol..

[32] Balermpas P., Michel Y., Wagenblast J., Seitz O., Weiss C., Rödel F., Rödel C., Fokas E. (2014). Tumour-infiltrating lymphocytes predict response to definitive chemoradiotherapy in head and neck cancer. Br. J. Cancer.

[33] Schuntermann D.B., Jaskolowski M., Reynolds N.M., Vargas-Rodriguez O. (2024). The central role of transfer RNAs in mistranslation. J. Biol. Chem..

[34] Stojanovic M.N., De Prada P., Landry D.W. (2000). Fluorescent sensors based on aptamer self-assembly. J. Am. Chem. Soc..

[35] Meng X., Li J., Wu Y., Cao X., Zhang Z. (2023). Rational design of hairpin aptamer using intrinsic disorder mechanism to enhance sensitivity of aptamer folding-based electrochemical sensor for tobramycin. Sens. Actuators B Chem..

[36] Li J., Xu T., Zheng Y., Liu D., Zhang C., Li J., Wang Z.A., Du Y. (2024). In silico study on a binding mechanism of ssDNA aptamers targeting glycosidic bond-containing small molecules. Anal. Chem..

[37] Pantsar T., Poso A. (2018). Binding affinity via docking: Fact and fiction. Molecules.

[38] Agu P.C., Afiukwa C.A., Orji O.U., Ezeh E.M., Ofoke I.H., Ogbu C.O., Ugwuja E.I., Aja P.M. (2023). Molecular docking as a tool for the discovery of molecular targets of nutraceuticals in diseases management. Sci. Rep..

[39] Castro-Alvarez A., Costa A.M., Vilarrasa J. (2017). The performance of several docking programs at reproducing protein–macrolide-like crystal structures. Molecules.

[40] Huang Y., Li Z., Hong Q., Zhou L., Ma Y., Hu Y., Xin J., Li T., Kong Z., Zheng Q. (2022). A stepwise docking molecular dynamics approach for simulating antibody recognition with substantial conformational changes. Comput. Struct. Biotechnol. J..

[41] Soremekun C., Jjingo D., Kateete D., Nash O., Grallert H., Peters A., Chikowore T., Batini C., Soremekun O., Fatumo S. (2024). Structural insights into conformational stability of both wild-type and mutant insulin receptor gene. Next Res..

[42] Liebl K., Zacharias M. (2023). The Development of Nucleic Acids Force Fields: From an Unchallenged Past to a Competitive Future. Biophys. J..

[43] Karsisiotis A.I., Da Silva M.W. (2012). Structural probes in quadruplex nucleic acid structure determination by NMR. Molecules.

[44] Minchin S., Lodge J. (2019). Understanding biochemistry: Structure and function of nucleic acids. Essays Biochem..

[45] Wang T., Chen C., Larcher L.M., Barrero R.A., Veedu R.N. (2019). Three decades of nucleic acid aptamer technologies: Lessons learned, progress and opportunities on aptamer development. Biotechnol. Adv..

[46] Raczyńska E.D., Sapuła M., Zientara-Rytter K., Kolczyńska K., Stępniewski T.M., Hallmann M. (2016). DFT studies on the favored and rare tautomers of neutral and redox cytosine. Struct. Chem..

[47] Wang X., Yu B., Iwahara J. (2022). Slow rotational dynamics of cytosine NH2 groups in double-stranded DNA. Biochemistry.

[48] Ropii B., Bethasari M., Anshori I., Koesoema A.P., Shalannanda W., Satriawan A., Setianingsih C., Akbar M.R., Aditama R., Fahmi F. (2024). The molecular interaction of six single-stranded DNA aptamers to cardiac troponin I revealed by docking and molecular dynamics simulation. PLoS ONE.

